# The benefit of adjuvant radiotherapy after breast conserving surgery in older patients with low risk breast cancer- a meta-analysis of randomized trials

**DOI:** 10.1186/s13014-017-0796-x

**Published:** 2017-03-23

**Authors:** Christiane Matuschek, Edwin Bölke, Jan Haussmann, Svjetlana Mohrmann, Carolin Nestle-Krämling, Peter Arne Gerber, Stefanie Corradini, Klaus Orth, Kai Kammers, Wilfried Budach

**Affiliations:** 1 Department of Radiation Oncology, Medical Faculty, Heinrich Heine University, Dusseldorf, Germany; 2 Department of Gynecologic and Obtresic, Heinrich Heine University, Dusseldorf, Germany; 3 Department of Senology, Sana Krankenhaus Dusseldorf, Dusseldorf, Germany; 4 Department of Dermatology, Medical Faculty Heinrich Heine University, Dusseldorf, Germany; 5 Department of Radiation Oncology, LMU Munich, Munich, Germany; 6 Department of General, Visceral and Thoracic Surgery, Harzkliniken Goslar, Goslar, Germany; 7 Division of Biostatistics and Bioinformatics, Department of Oncology, The Sidney Kimmel Comprehensive Cancer Center at Johns Hopkins, The Johns Hopkins University School of Medicine, Baltimore, MD USA; 8 Klinik für Strahlentherapie und Radiologische Onkologie, Heinrich Heine Universität, Moorenstr. 5, D-40225 Düsseldorf, Germany

**Keywords:** Radiation therapy, Tamoxifen, Low risk breast cancer, Adjuvant therapy

## Abstract

**Purpose/Objective(s):**

It is currently unclear whether patients with low risk breast cancer receiving adjuvant endocrine therapy need adjuvant radiation therapy after breast conserving surgery. The data of randomized trials are available.

**Materials/Methods:**

In a database search 5 randomized trials including in total 3766 mostly elderly patients with early stage breast cancer tr﻿eated either with adjuvant endocrine therapy or with endocrine therapy and additional whole breast radiation after breast conserving surgery were identified. Published hazard ratios for time to local recurrence were the basis of our meta-analysis. Meta-analysis of the effect sizes on local recurrence was performed using a random effects model based on parameter estimates of log hazard ratios in Cox models and their standard errors. Furthermore, overall survival was examined.

**Results:**

Adjuvant hormone therapy alone in mostly older patients with low risk breast cancer resulted in significantly shorter time to local relapse compared to radiation therapy combined with hormone therapy (Hazard Ratio: 6.8, 95% CI: 4.23–10.93, *p* < 0.0001) . There was no significant difference for overall survival.

**Conclusion:**

Additional radiation therapy to hormone therapy did improve local relapse in breast cancer patients but did not show significant impact on overall survival.

## Introduction

Randomized studies provide evidence that breast conserving surgery (BCS) combined with postoperative radiation therapy (RT) results in long-term overall survival comparable to modified radical mastectomy [1–3]. Postoperative RT leads to a significant reduction in local relapse compared to BCS alone [2, 3]. The recent meta-analysis of the Early Breast Cancer Trialists Collaborative Group (EBCTCG) showed that the addition of RT after BCS is associated with a large survival advantage in node positive breast cancer, but only a small survival advantage in node negative breast [1].

For most elderly women with early stage breast cancer, the current standard treatment after BCS is adjuvant whole-breast RT and adjuvant endocrine treatment [4–7].

The question whether RT can be safely omitted in women with low risk breast cancer is still under debate [8–11]. Some physicians believe that combining hormonal therapy with RT results in relevant late side effects without providing significant benefit and therefore should be abandoned. On the other hand, published data outline RT as a highly effective therapy in reducing local recurrence rates. Therefore, we performed a meta-analysis to investigate the importance of additional adjuvant RT in patients ﻿with low risk breast cancer.

## Patients and methods

The aim of this meta-analysis was to investigate the impact of adjuvant radiotherapy on breast cancer patients with low risk of recurrence. Low risk was defined as tumor size <3 cm, N0, estrogene or progesterone receptor positive disease in postmenopausal women (age >50 years). A PubMed research with the search term “breast and radiotherapy and (tamoxifen OR endocrine)” restricted to “randomized trials was performed. There was no ethics approval necessary because in this meta-analysis we were retreiving numbers from the published manuscripts and pooling results. Out of 218 matches 8 randomized controlled trials were identified that compared sole adjuvant endocrine therapy to adjuvant endocrine therapy and adjuvant radiotherapy [12–14]. One additional trial [15] was identified by screening references of published trials and all important cancer meetings like ASCO, ASTRO, ESTRO and ECCO. Three trials were excluded, because of the inclusion of high risk patients (node positive) and one trial could not be included, because of inadequate reporting of the end points local relapse and overall survival. In summary, we were able to identify a total of five randomized prospective trials [8, 9, 11, 16, 17] that fulfilled the entry criteria. The main characteristics of these trials are shown in Table 1. Endpoints of the meta-analysis were local tumor relapse and death from any cause. Because of insufficient reporting in the majority of the identified trials, we were unable to analyze data on disease free survival and distant metastases free as additional endpoints.

**Table 1:** study characteristics

	Fyles et al. NEJM 2004	Hughes et al. CO 2013	Pötter et al. Int J radiation Oncol Biol Phys 2007	Blamey et al. Eur J Cancer 2013	Kunkler Lancet Oncol 2015
Trial design	Multicenter, randomized	Multicenter, randomized	Multicenter, randomized	Multicenter, randomized	Multicenter, randomized
Number of patients recruitment	769 1992–2000	636 1994–1999	869 1996–2004	204 1992–2000	1326 2003–2009
Inclusion criteria: age	≥50 years	≥70 years	≥50 years^a^	≥50 years	≥65 years
Tumor diameter	<5 cm	<2 cm	<3 cm	<2 cm	<3 cm
ER/PR positiv	100%	100%	100%	100%	100%
Her-2neu	unknown	unknown	unknown	unknown	Unknown, because this marker was not routinely assessed at the beginning of the trial
Other main inclusion criteria	Postmenopausal status, MO	Postmenopausal status, MO	Postmenopausal status, MO	Postmenopausal status, MO	Postmenopausal status, MO
Breast surgery	lumpectomy	lumpectomy	lumpectomy	Wide local excision	lumpectomy

^a^exeption 3 patients, minimum age 46 years

In our meta-analysis a total of 3766 mostly elderly women from five randomized trials with early stage breast cancer were included to assess the potential benefit for the addition of whole breast RT to endocrine therapy after BCS. Analyzed end points were local recurrence and overall survival. Meta-analysis of the effect sizes on time to local recurrence was performed by using a random effects model based on parameter estimates of log hazard ratios in Cox models and their standard errors. Neither hazard ratios for overall survival nor survival curves were available from most trials. However, we were able to estimate odds ratios for death from any cause and their confidence limits using published event rates at 5 or 10 years, recruitment time, and median follow up. Meta-analysis of the effect sizes on death from any cause was performed using a random effects model based on parameter estimates of log odds ratios and their standard errors. Results are presented with forest plots in which the effect size estimates of all single studies and their combined estimate are visualized. Horizontal bars indicate the amount of variation (95% confidence intervals of the parameter estimates).

## Results

In a Canadian study [17] the authors investigated the effect of breast irradiation plus tamoxifen on disease-free survival and time to local relapse in women 50 years of age or older with T1 or T2 node-negative breast cancer. A total of 769 women with early breast cancer (tumor diameter, 5 cm or less) were included in their trial and randomly assigned to receive breast irradiation plus tamoxifen (386 women) or tamoxifen alone (383 women). The median follow-up time was 5.6 years. The rate of local relapse at 5 years was 7.7% in the tamoxifen group and 0.6% in the group given tamoxifen plus irradiation (HR: 9.02; 95% CI: 3.52-23.02; *p* < 0.001), with corresponding 5-year disease-free survival rates of 84 and 91% (*p* = 0.004). A subgroup analysis of 611 women with T1, receptor-positive tumors indicated a benefit from RT (5-year rates of local relapse, 0.4% with tamoxifen plus RT and 5.9% with tamoxifen alone; *p* < 0.001). In their study they demonstrated a significant difference in the rate of axillary relapse at 5 years (2.5% in the tamoxifen group and 0.5% in the group given tamoxifen plus irradiation, *p* = 0.049), but no significant difference in the rates of distant relapse or overall survival (OS).

The second study which was included in our meta-analysis was performed by the Harvard Medical School in Boston [9]. The authors investigated a benefit for adjuvant RT after breast-conserving surgery and tamoxifen in women age >/= 70 years with early-stage breast cancer. A total of 636 women (age >/= 70 years) with clinical stage I (T1N0M0 according to TNM classification) estrogen receptor (ER)-positive breast carcinoma treated by lumpectomy were randomly assigned to receive tamoxifen plus RT (TamRT; 317 women) or tamoxifen alone (Tam; 319 women). Primary end points were time to locoregional recurrence, frequency of mastectomy, breast cancer-specific survival, time to distant metastasis and OS. Median follow-up was 12.6 years. At 10 years, 98% of patients receiving TamRT (95% CI, 96 to 99%) compared with 90% of those receiving Tam (95% CI, 85 to 93%) were free from local and regional recurrences. As compared with the Tam group, the TamRT group experienced a significantly longer time to locoregional recurrence (HR: 0.18; 95% CI: 0.07-0.42; *p* < ﻿0.001). There were no significant differences in time to mastectomy, time to distant metastasis, breast cancer-specific survival, or OS between the two groups. Ten-year OS was 67% (95% CI, 62 to 72%) and 66% (95% CI, 61 to 71%) in the TamRT and Tam groups, respectively.

A study from Vienna and the Austrian Breast Cancer Group [11] was designed to randomly assign 869 women (38 women were ineligible) to receive breast RT +/− boost (*n* = 414) or not (*n* = 417) after BCS (ABCSG Study 8A). Favorable early breast cancer was specified as tumor size <3 cm, Grading 1 or 2, negative lymph nodes, positive estrogen and/or progesterone receptor status, and manageable by BCS. Breast RT was performed after lumpectomy with two tangential opposed breast fields with mean 50 Gy, plus boost in 71% of patients with a mean of 10 Gy, in a median of 6 weeks. The primary endpoint was local relapse-free survival; further endpoints were contralateral breast cancer, distant metastases, as well as disease-free and OS. The median follow-up was 53.8 months. The mean age was 66 years. Overall, there were 21 local relapses, with two relapses in the RT group (5-y rate 0.4%) vs. 19 in the no-RT group (5.1%) (HR:10.21, (95% CI: 2.38–43.84); *p* = 0.002). Overall relapses occurred in 30 patients, with seven events in the RT group (5-y rate 2.1%) vs. 23 events in the no-RT group (6.1%) (HR: 3.5, *p*=0.002). No significant differences were found for distant metastases and OS.

Furthermore we identified a trial from England which was published in the European Journal of Cancer [8]. Patients with primary invasive breast cancer <2 cm diameter, grade 1 or good prognosis special type, and node negative, treated by wide local excision (WLE) with clear margins were randomized into a 2 × 2 clinical trial of factorial design with or without RT and with or without tamoxifen. Trial entry was allowed to either comparison or both. The actuarial breast cancer specific survival in 1135 randomized patients at 10 years was 96%. Analysis by intention to treat showed that LR after WLE was reduced in patients randomized to RT (HR 0.37, CI 0.22–0.61 *p* < 0.001) and to tamoxifen (HR 0.33, CI 0.15 - 0.70 *p* < 0.004). Actuarial analysis of patients entered into the four-way randomization showed that LR after WLE alone was 1.9% per annum (pa) versus 0.7% with RT alone and 0.8% with tamoxifen alone. No patient randomized to both adjuvant treatments developed LR. Analysis by treatment received showed LR at 2.2% pa for surgery alone versus 0.8% for either adjuvant RT or tamoxifen and 0.2% for both treatments. Comparison between tamoxifen alone (*n*=106) and tamoxifen+RT (*n*=98) resulted in a significant reduction of local relapse in the tamoxifen+RT group (HR: 7.34, 95% CI: 1.79-30.1; *p*=0.006).

Finally, the recently published results of the PRIME II trial were included in our meta-analysis [16]. A total of 1326 women aged 65 years or older with early breast cancer judged low-risk (i.e., hormone receptor-positive, axillary node-negative, T1-T2 up to 3 cm at the longest dimension, and clear margins; grade 3 tumor histology or lymphovascular invasion, but not both, were permitted), who underwent BCS and were receiving adjuvant endocrine treatment, were recruited into a phase 3 randomized controlled trial at 76 centers in four countries. Eligible patients were randomly assigned to either whole-breast RT (40–50 Gy in 15–25 fractions) or no RT by computer-generated permuted block randomization, stratified by center, with a block size of four. The primary endpoint was ipsilateral breast tumor recurrence. Women who had undergone BCS and who were receiving adjuvant endocrine treatment were randomly assigned to receive whole-breast irradiation and 668 were allocated to no further treatment. After median follow-up of 5 years (IQR 3.84–6.05), ipsilateral breast tumor recurrence was 1.3% (95% CI 0.2–2.3; *n* = 5) in women assigned to whole-breast RT and 4.1% (95% CI 2.4–5.7; *n* = 26) in those assigned no RT (*p* = 0.0002). Compared with women allocated to whole-breast RT, the univariate hazard ratio for ipsilateral breast tumor recurrence in women assigned to no RT was 5.19 (95% CI 1.99–13.52; *p* = 0.001). No differences in regional recurrence, distant metastases, contralateral breast cancers, or new breast cancers were noted between groups. Five-year OS was 93.9% (95% CI 91.8–96.0) in both groups (*p* = 0.34); 89 women died; eight of 49 patients allocated to no RT and four of 40 assigned to RT died from breast cancer.

**Fig 1:**
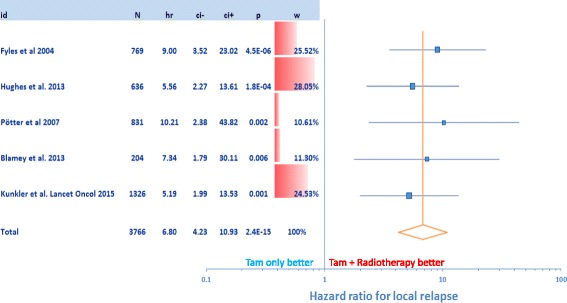
Hazard ratios for local relapse Results are presented with forest plots in which the estimates of the hazard ratios of all single studies and their combined estimate are visualized. *Horizontal bars* indicate the amount of variation (95% confidence intervals of the parameter estimates)

**Fig. 2:**
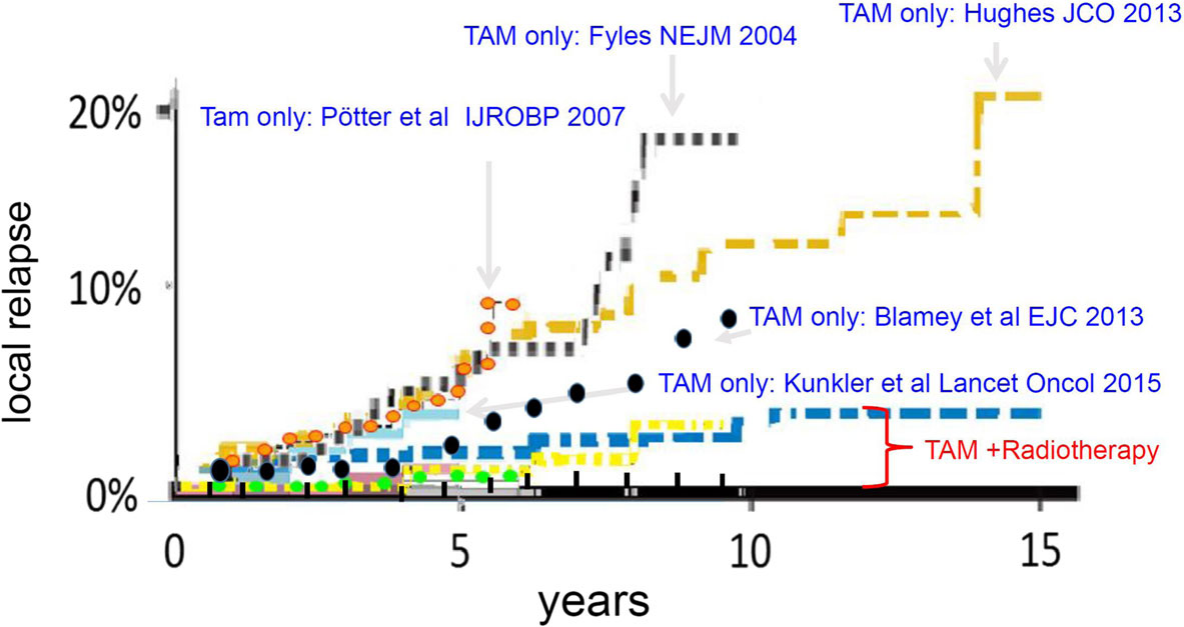
Time to local recurrence. *Blue letters*: treatment arm with Tamoxifen (Tam) only without RT. *Red letters*: TAM + RT

Tamoxifen without RT results in an increase of local relapse (Figs. 1 and 2). After 10 to 20 years there is a significantly higher risk of up to 20% that patients develop a local relapse. Patients receiving RT and endocrine therapy have a very low local relapse rate of less than 5%. We found that treatment with tamoxifen alone in older patients and low risk breast cancer did result in shorter time to local relapse compared to a combination of both therapeutic strategies (HR: 6.8, 95% CI: 4.23–10.93, *p* < 0.0001).

**Fig. 3:**
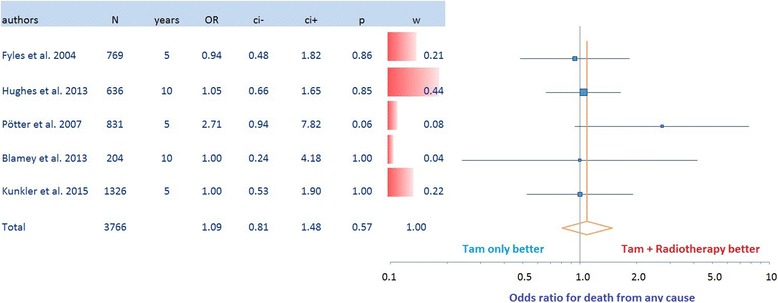
Odds ratio for death to any course. Results are presented with Odds ratio with confidence intervals in forest plots. *Horizontal bars* indicate the amount of variation (95% confidence intervals of the parameter estimates)

For death from any cause, neither in the individual trials nor in the meta-analysis statistically significant differences between treatments were observed (Odds ratio 1.09, 95% CI 0.81–1.48, *p* = 0.57) (Fig. 3). At 5- and 10-year overall survival were within the expected range of an age matched US general population [18]. Using the 95% CI of the odds ratio of the meta-analysis, neither a survival benefit for the addition of radiotherapy to endocrine therapy of 3% at 5 years and 7% at 10 years nor a survival disadvantage of 1% at 5 years and 2% at 10 years can be excluded.

### Side effects

Side effects were reported from the Canadian trial by Fyles et al [17]. They report that adverse events according to CTC-criteria did not differ significantly between tamoxifen plus RT and tamoxifen alone (*n* = 39 in the Tamoxifen and RT group and *n* = 30 in the Tamoxifen alone group, *p* = 0.33). In a subgroup of this trial Rayan et al [19]. investigated the impact of RT on breast pain and quality of life and found no adverse influence of irradiation [20].

In a subgroup analysis of the PRIME II trial Williams et al [21] reported that omission of radiation therapy did not improve quality of life after 5 years in a very detailed EORTC QLC-C30 and EORTC QLC-B23 questionnaire. Only insomnia was significantly more frequent in the patient group with Tamoxifen alone. Some differences were apparent with subscales of the questionnaires. Two patients in the Tamoxifen plus RT group developed fibrosis °III, whereas few patients (number of patients not published) developed fibrosis °II in the Tamoxifen alone group. Retraction/atrophy °II occurred similar in both groups (number of patients not published), whereas °III developed in 1 patient in the Tamoxifen plus RT group.

In, summary the authors conclude that RT is well tolerated and does not impair quality of life.

A detailed overview of the side effects and quality of life questionnaire of all trials is shown in Table 2.

**Table 2:** Side effects and quality of life

Trial	Fyles et al.		Subgroup analysis (Fyles et al.): “breast pain study” published by Rayan et al.		Kunkler et al. PRIME II trial subgroup analysis (Williams et al.)		Blamey et al.		Pötter et al.		Hughes et al.	
n=	Tam *n* = 383	Tam + RT *n* = 386	Tam *n* = 45	Tam + RT *n* = 41	Tam *n* = 128	Tam + RT *n* = 127	Tam	Tam + RT	Tam	Tam + RT	Tam	Tam + RT
Erythema ≥ °III	0	4	s. QLC	s. QLC	n.r.		n.r.		n.r.		n.r.	
Edema ≥ °III	1	2	s. QLC	s. QLC	0	1	n.r.		n.r.		n.r.	
Fatique ≥ °III	0	4	s. QLC	s. QLC	n.s.		n.r.		n.r.		n.r.	
Teleangiectasia ≥ °III	n.r.	n.r.	s. QLC	s. QLC			n.r.		n.r.		n.r.	
Fibrosis ≥ °III	n.r.	n.r.	s. QLC	s. QLC	few patients °II in the Tam alone goup, 2 patients °III in the Tam + RT group		n.r.		n.r.		n.r.	
Retraction/Atrophy	n.r.	n.r.	s. QLC	s. QLC	°II similar in both groups, °III: *n*= 1 patient in the Tam + RT group		n.r.		n.r.		n.r.	
Ulcer	n.r.	n.r.	s. QLC	s. QLC	*n*= 2 in both groups		n.r.		n.r.		n.r.	
Pain management	n.r.	n.r.	s. QLC	s. QLC	°III: *n* = 1 in the Tam alone group		n.r.		n.r.		n.r.	
Lung toxicity	n.r.	n.r.	s. QLC	s. QLC	n.r.		n.r.		n.r.		n.r.	
Cardiac Toxicity	n.r.	n.r.	s. QLC	s. QLC	n.r.		n.r.		n.r.		n.r.	
Depression	1	2	s. QLC	s. QLC	n.s.		n.r.		n.r.		n.r.	
Hot flashes	23	30	s. QLC	s. QLC	n.s.		n.r.		n.r.		n.r.	
Other		Stroke *n* = 1	s. QLC	s. QLC	n.r.		n.r.		n.r.		n.r.	
EORT QLC-C30 scale	n.r.		n.s.	n.s.	n.s. (except insomnia: significant more frequently in the Tam alone group)		n.r.		n.r.		n.r.	
EORT QLC-B23 scale	n.r.		n.s.	n.s.	n.s.		n.r.		n.r.		n.r.	
Contraleteral breast cancer	10	10	s. QLC	s. QLC	n.r.		n.r.		n.r.		n.r.	
Second cancers	41	31	n.a.	n.a.	n.r.		n.r.		n.r.		n.r.	
∑ side effects	30	39			n.r.		n.r.		n.r.		n.r.	
∑ *p*-value	*P* = 0.33				n.s.		n.r.		n.r.		n.r.	

*n.r.: not reported, *n.s.: not significant, *n.a.: not applicable

## Discussion

There is a large debate on the role of RT in elderly women with low risk breast cancer. The results of large randomized trials are summarized in this meta-analysis. Of note, this meta-analysis is limited by the absence of individual patient data, which were not available.

Radiation in addition to endocrine treatment following BCS in low risk breast cancer patients (<3 cm diameter, hormone receptor positive, N0, postmenopausal) substantially reduces the hazard to develop a local recurrence by a factor of 6.8 (CI 4.4–11.9; *p* < 0,001) (Fig. 2). However, additional radiation did not translate into a significant improvement in OS in this group [8–11, 16, 17, 22, 23], although the available data do not exclude a survival advantage of 3% at 5 years and up to 7% at 10 years. The absolute risk for local recurrence with endocrine therapy alone is quite low at 5 years (<5%), but increases continuously to about 10% at 10 years with no recognisable plateau thereafter in the only trial with long term follow to 15 years [10]. The rate of secondary mastectomies has been reported from two trials indicating a 4.4 – 6.0 fold risk [Kunkler et al.: 1.8% (12/668) vs. 0.3% (2/658), *p* > 0.05.; Hughes et al.: 8.5% (27/319) vs. 2% (6/315), *p* < 0.05], if radiotherapy is omitted. It remains unclear how patients should be advised in clinical practise in view of the large risk reduction by radiotherapy in terms of local relapse, but no proven advantage in terms of overall survival. Obviously, omission of radiotherapy is quite safe in low risk breast cancer patients with limited life expectancy below 5 years. As most patients will present with substantially longer life expectancies individual counseling about the risks and benefits of radiotherapy is strongly recommended. Detailed data on toxicities are not available from the trials included in this meta-analysis. However, acute and long term toxicity of adjuvant radiotherapy using hypofractionated radiotherapy schedules like 15× 2.66 Gy in 3 weeks is quite limited, if modern radiation techniques are used [24]. Therefore, radiotherapy is a reasonable option especially for patients with low risk breast cancer having life expectancies greater than 10 years.

Further subgroup analyses could help to identify patients who may not substantially benefit form radiotherapy. In this regard, it is important to mention one of the limitations of the present meta-analysis as we had no access to individual patients data and were consequently unable to look for such subgroups. Another weakness of the available data and consequently the presented meta-analysis is the unknown Her2-status in all studies. Liu et al. [20] retrospectivly analysed a number of molecular markers (HER2, CK5/6, EGFR, Ki-67) from a part of the patients in the Canadian trial [17]. They found that patients with luminal A and B like breast cancer had a small benefit from radiotherapy in addition to endocrine treatment (luminal A: HR = 0.4, *p* = 0.11; luminal B HR = 0.51, *p* = 0.18), whereas patients with Her2-positive and triple negative disease had a larger advantage from radiotherapy in terms of locoregional relapse (HER2-positive/“triple negative”: HR = 0.13, *p* = 0.0015). According to these observations omission of radiotherapy would be safe in patients above 70 years with small node negative, luminal A breast cancer. However, independent prospective confirmatory data are required.

Regarding side effects the authors of the mentioned trials conclude that radiation therapy is well tolerated without excess toxicity. Side effects as well as the quality of life (QLC-C30 and QLC-B23) did not differ significantly between the irradiated groups and the patient groups receiving Tamoxifen alone. Of note, the trials performed by Blamey, Kunkler and Pötter did not publish information on side effects.

Another question is whether endocrine treatment is needed after BCS in patients above 70 years with small node negative breast cancer, if adjuvant radiotherapy is administered. According to the results of two randomized trials [8, 14] sole adjuvant endocrine therapy and sole adjuvant radiotherapy are equivalent regarding all important oncological endpoints. Endocrine therapy is frequently associated withfatigue symptoms and possible severe side effects, as thromboembolic events and endometrial cancer related to tamoxifen as well as osteoporosis and arthralgia related to aromatase inhibitors. In view of the at least 5 years lasting endocrine the typical side effects of a three weeks hypofractionated adjuvant radiotherapy like faint erythema and minor edema appear relatively moderated and of short duration.

Another discussion is whether partial breast radiotherapy could represent an alternative treatment option for low risk breast cancer patients and result in comparable local control rates as whole breast radiotherapy. In a recently published meta-analysis (Marta et al. 2015) of eight randomized trials (*n* = 8653) comparing whole breast radiotherapy with partial breast radiotherapy a significantly higher rate of in-breast recurrences was reported for partial breast radiotherapy (HR = 4.54, 95% CI: 1.78–11.61, *p* = 0.002). Interestingly, the hazard in favour for whole breast radiation vs. partial breast radiation is in the same range as in the meta-analysis reported here (HR=6.8) for the comparison of whole breast radiotherapy to no radiotherapy (endocrine treatment in all patients in both comparisons), raising the question, whether partial breast radiotherapy is any better than no radiotherapy at all. In the absence of direct randomized comparisons and in view of the different characteristics of patients included in these two meta-analyses, the question cannot conclusively be answered.

## Conclusions

In summary adjuvant RT in addition to standard endocrine therapy in low risk breast cancer patients was not associated with a significantly improved overall survival, but reduced the hazard of local recurrence substantially by a factor of 6.8 corresponding to an absolute decrease in local recurrence of 3–5% at 5 years and 9–14% 10 years. Individual counselling is of high clinical relevance in this situation. However in view of the relatively low toxicity of modern radiotherapy, adjuvant radiotherapy should be advised in patients with life expectancies larger than 5–10 years.

## Abbreviations

ASCO: American Society of Clinical Oncology
ASTRO: American Society of Radiation Oncology
BCS: Breast conserving surgery
CI: Confidence interval
EBCTCG: Early Breast Cancer Trialists
ECCO: European Cancer Organisation
ESTRO: European Society for Radiotherapy and Oncology
GY: Gray
HER: Human epithelial growth factor
HR: Hazard ratio
LR: Local relapse
N: Node
OS: Overall survival
Pa: Per annum
RT: Radiotherapy
Tam: Tamoxifen
Vs: versus
WLE: Wide local excision
Y: Year
